# Drivers of the US CO_2_ emissions 1997–2013

**DOI:** 10.1038/ncomms8714

**Published:** 2015-07-21

**Authors:** Kuishuang Feng, Steven J. Davis, Laixiang Sun, Klaus Hubacek

**Affiliations:** 1 Department of Geographical Sciences, University of Maryland, College Park, Maryland 20742, USA; 2 Department of Earth System Science, University of California, Irvine, Irvine, California 92697, USA; 3 Institute of Applied Ecology, Chinese Academy of Sciences, Shenyang 110016, China; 4 Department of Financial and Management Studies, SOAS, University of London, London WC1H 0XG, UK; 5 International Institute for Applied Systems Analysis (IIASA), A-2361 Laxenburg, Austria

## Abstract

Fossil fuel CO_2_ emissions in the United States decreased by ∼11% between 2007 and 2013, from 6,023 to 5,377 Mt. This decline has been widely attributed to a shift from the use of coal to natural gas in US electricity production. However, the factors driving the decline have not been quantitatively evaluated; the role of natural gas in the decline therefore remains speculative. Here we analyse the factors affecting US emissions from 1997 to 2013. Before 2007, rising emissions were primarily driven by economic growth. After 2007, decreasing emissions were largely a result of economic recession with changes in fuel mix (for example, substitution of natural gas for coal) playing a comparatively minor role. Energy–climate policies may, therefore, be necessary to lock-in the recent emissions reductions and drive further decarbonization of the energy system as the US economy recovers and grows.

## Abstract (web summary)

US CO_2_ emissions dropped ∼11% between 2007 and 2013; a trend widely attributed to the increased use of natural gas over coal, yet the drivers behind this decline remain unquantified. Here, the authors analyse the drivers and show that the recent economic downturn is primarily responsible for the emissions drop.

## Graphical abstract

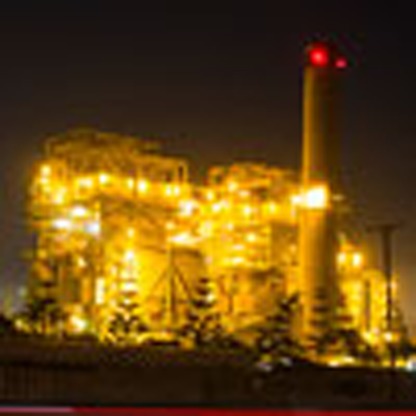

The CO_2_ emissions from the burning of fossil fuels are the primary cause of anthropogenic climate change^1^, and the United States emits more CO_2_ each year than any other country except China. In the decade before 2007, US CO_2_ emissions grew by an average 0.7% per year. However, beginning in 2007, US emissions decreased, reaching a minimum of 5,284 Mt CO_2_ in 2012—12% lower than 2007 levels and 5% lower than 1997 levels^2^. This recent decline is good news and is consistent with the Obama administration's stated goal of reducing CO_2_ emissions by 17% in 2020 and 83% in 2050 relative to 2005 levels^3^. Assuming no change in emissions outside the power sector, the new rules proposed by the US Environmental Protection Agency in June 2014 to limit CO_2_ emissions from power plants will require US emissions to decrease to 4,200 Mt CO_2_ in 2030—a further 20% reduction from 2013 levels^4^.

Coinciding with the post-2007 decline in emissions, innovations in hydraulic fracturing technology have dramatically increased domestic supplies of gas^5^,^6^. Commentators in the scientific community and media have linked the two trends, celebrating the climate benefits of the gas boom^7^,^8^,^9^. Recently, the Third National Climate Assessment of the United States Global Change Research Program also adopted this conclusion, stating that the decrease in US CO_2_ emissions was ‘…largely due to a shift from coal to less CO_2_-intensive natural gas for electricity production'^10^. Yet, despite potentially significant implications for US climate and energy policy, there has been no quantitative analysis of whether the gas boom and changes in the fuel mix of the power sector are indeed driving the decrease in US CO_2_ emissions.

Here, we use input–output structural decomposition analysis (SDA) to assess sources of change in US CO_2_ emissions over a decade of mostly increasing emissions, 1997–2007, and then over the period of mostly decreasing emissions, 2007–2013. Our analysis quantifies the contribution of six different factors to changes in US emissions. These factors are: population growth; changes in consumption volume caused exclusively by changes in per capita consumption of goods and services; shifts in consumption patterns or the types of goods and services being consumed; adjustments in production structure or the mix of inputs (for example, labour, domestic and imported materials) required to produce US goods and services; changes in fuel mix as reflected by the CO_2_ emitted per unit of energy used; and changes in energy intensity or the energy used per inflation-adjusted unit of economic output. The SDA in this research is based on the additive decomposition of the changes in emission determined by six multiplicative factors acting as accelerators or retardants of the emission dynamics. Each term in the decomposition is a product of the change in one explicative factor and the level values of the other five factors, and thus represents the contribution of one explicative factor to the total change in emission. For example, in the term where population is the explicative factor, the values of consumption volume, production structure, consumption patterns, energy intensity and fuel mix are held unchanged and only population varies. In this way, the SDA method allows us to quantify the contribution of each of the assessed factors to the trend in emissions. Details of our methodology and data sources are in the Methods section (including Supplementary Methods). We find that before 2007, rising emissions were driven by economic growth: 71% of the increase between 1997 and 2007 was due to increases in US consumption of goods and services, with the remainder of the increase due to population growth. Concurrent with the global economic recession, 83% of the decrease during 2007–2009 was due to decreased consumption and changes in the production structure of the US economy, with just 17% related to changes in the fuel mix. During the economic recovery, 2009–2013, the decrease in US emissions has been small (<1%), with nearly equal contributions from changes in the fuel mix, decreases in energy use per unit of GDP, changes in US production structure, and changes in consumption patterns. We conclude that substitution of gas for coal has had a relatively minor role in the emissions reduction of US CO_2_ emissions since 2007.

## Results

### Growing emissions from 1997 to 2007

**Figure 1:**
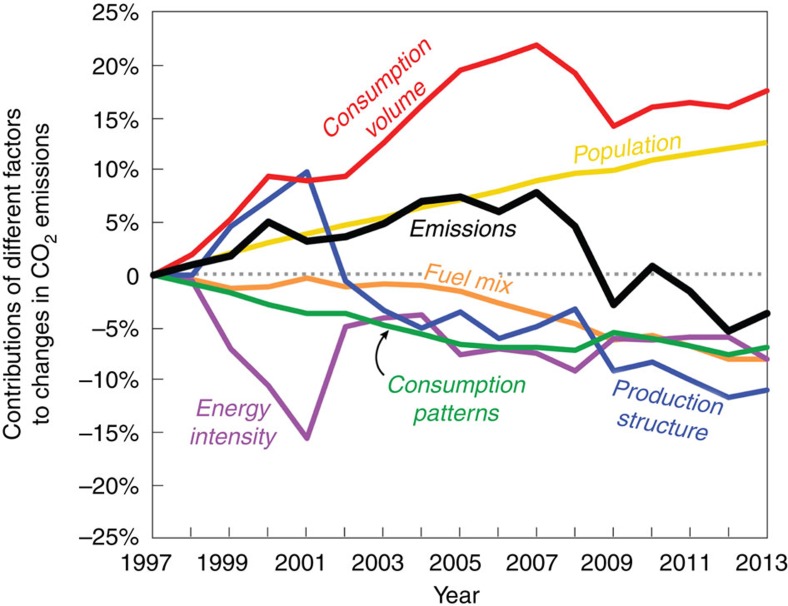
Contributions of different factors to changes in the US CO_2_ emissions between 1997 and 2013. Using 1997 as base year, the solid black line shows the percentage change in total CO_2_ emissions. The other lines show the contribution to the change in emissions from consumption volume (red), population (yellow), consumption patterns (green), production structure (blue), energy intensity (purple) and fuel mix (orange).

Between 1997 and 2007, US emissions increased by 7.3% (Fig. 1, black curve). Our analysis shows that the main factor behind this increase was an increase in consumption volume caused by growth in per capita consumption of goods and services in the United States. Indeed, increases in such consumption volume correspond to a contribution of a 21.8% increase in emissions over this decade (Fig. 1, red curve). The next most important factor influencing CO_2_ emissions over the same period was population growth. Immigration and natural growth have resulted in steady population growth at a rate of ∼1% per year since 1997. These population gains contributed to an 8.9% increase in emissions between 1997 and 2007 (Fig. 1, yellow curve).

**Figure 2:**
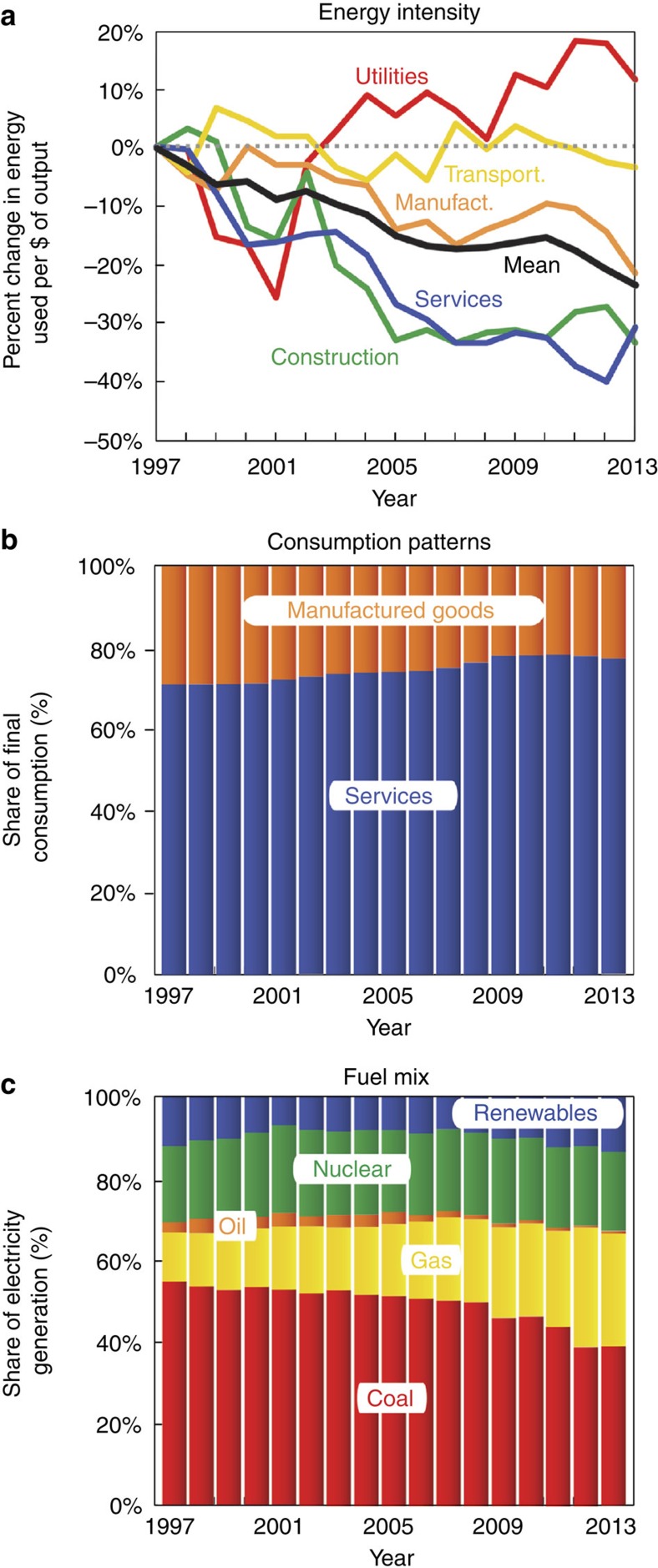
Trends underlying the decomposed factors. (**a**) Per cent changes in the energy intensity (energy used per dollar (US$) of output) of key sectors in the US economy, (**b**) shares of final demand made up of manufactured goods (that is, food, clothing, agriculture, paper and printing, chemical manufacture (manufact.), petroleum refining, metal manufacturing, machinery and equipment, utilities and construction) and services (that is, retail, hotel, transport, shipping, real estate, public administration, defense, education, health, community and social work, and household employment.) and (**c**) changes in the fuel mix of the US electricity sector.

However, other factors slowed the growth of emissions between 1997 and 2007: decreases in the energy intensity of GDP; changes in the consumption patterns of US consumers; shifts in production structure; and decreases in the use of coal as an energy source. For instance, over this period, the energy used per dollar of economic output decreased by 17% (Fig. 2a, black curve), the share of consumer spending on manufactured goods decreased by ∼4% (Fig. 2b), the share of imported inputs to the US industry sectors increased (for example, imports to petroleum and coal products sector increased by 6.7%, and imports to the chemical products, primary metals and textile sectors increased by 2.7%, 2.5% and 2.1%, respectively)^11^, and the share of US electricity generated from coal decreased by ∼5% while the share generated from natural gas increased by 8% (Fig. 2c). All of these trends exerted a downward influence on emissions. Between 1997 and 2007, changes in energy intensity, consumption patterns, production structure and fuel mix contributed to retarding emissions of 7.4, 6.9, 4.9 and 3.6%, respectively (Fig. 1, purple, green, blue and orange curves, respectively).

### Declining emissions from 2007 to 2013

US CO_2_ emissions stopped growing in 2007, and decreased by ∼11% between 2007 and 2013 (Fig. 1, black curve). Looking at this time period in aggregate, the only factor which acted to increase emissions over the period was continued and steady population growth (+3.7%) (Fig. 1, yellow curve). However, the upward influence of population growth was overwhelmed by the downward influence of changes in production structure (−6.1%), fuel mix (−4.4%), consumption volumes triggered by per capita consumption (−3.9%), energy intensity of GDP (−0.5%) and changing consumption patterns (−0.4%; Fig. 1, blue, orange, red, purple and green curves, respectively).

**Figure 3:**
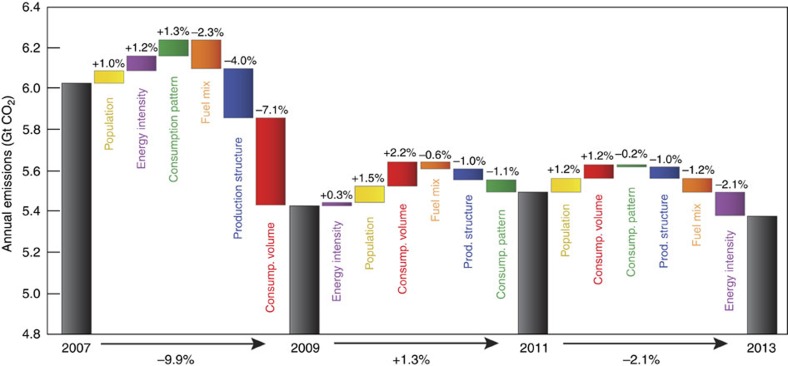
Contributions of different factors to the decline in US CO_2_ emissions 2007–2009 and 2009–2011 and 2011–2013. Between 2007 and 2009, decreases in the volume of goods and services consumed during the economic recession (red) was the primary contributor to the nearly 10% drop in emissions. But between 2009 and 2011, consumption (consump.) volume rebounded, population grew and the energy intensity of output increased, driving up emissions by 1.3% against modest decreases in the carbon intensity of the fuel mix and shifts in production structure and consumption patterns. Between 2011 and 2013, increases in population and consumption volume again pushed emissions upward, but overall emissions decreased by 2.1% due to further changes in production (prod.) structure, consumption patterns, decreasing use of coal and decreases in energy intensity of output. Not shown here, emissions increased by 1.7% between 2012 and 2013, driven primarily by increases in consumption volume.

Although all of the analysed factors except population contributed to the decrease in emissions during 2007–2013, different factors dominated over shorter periods. Figure 3 subdivides 2007-2013 into 2-year periods, showing that emissions fell by 9.9% from 2007 to 2009, increased by 1.3% between 2009 to 2011 and decreased again by 2.1% between 2011 and 2013.

**Figure 4:**
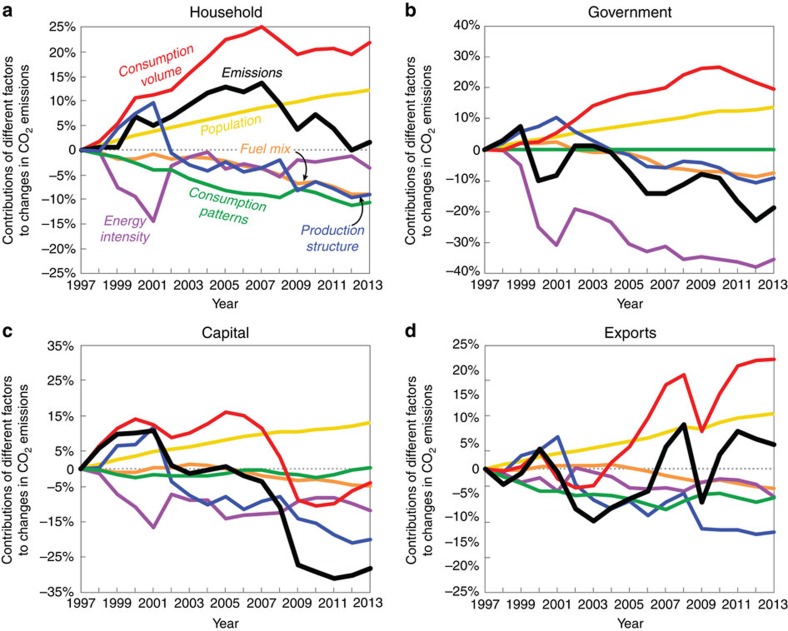
Contributions of different factors to changes in US CO_2_ emissions specific to different final demand components 1997–2013. Shown are changes in emissions related to household expenditures (**a**), government expenditures (**b**), capital investment (**c**) and exports (**d**). In each panel, the solid black line shows the percentage change in CO_2_ emissions triggered by changes in the corresponding final demand component, and the other lines show the contribution to the change in emissions from consumption volume (red), population (yellow), consumption patterns (green), production structure (blue), fuel mix (orange) and energy intensity (purple).

More than half (53%) of the initial and most substantial decrease in emissions, between 2007 and 2009, was due to a sharp drop in the volume of consumed goods as a result of reduction in per capita consumption during the global economic recession (Fig. 3, red bar). In particular, Fig. 4 shows that sharp decreases in the volume of capital expenditures and exported goods between 2007 and 2009 drove down associated emissions by 25% and 18%, respectively. Changes in the production structure of the US economy (that is, the volume and type of intermediate goods demanded) and the fuel mix of the energy sector contributed 30% and 17% of the initial (2007–2009) decrease in emissions, respectively, while increases in the energy intensity of the US economy and changing consumption patterns exerted modest upward influences on emissions during the same period.

As the US economy had slowly recovered from the global economic recession, between 2009 and 2013, the average annual change in US emissions was small: a 0.2% decrease. Economic recovery is reflected by the upward influence of the volume of goods consumed on emissions during both 2009–2011 and 2011–2013. Between 2009 and 2011, rising consumption volume, population growth, and increasing energy intensity urged emissions up by a combined 4.0% (2.2%, 1.5% and 0.3%, respectively), which was only partly offset by the changes in consumption patterns (−1.1%), production structure (−1.0%) and fuel mix (−0.6%), resulting in an actual increase in emissions of 1.3% (Fig. 3). However, between 2011 and 2013, the upward influence of consumption volume and population on emissions was less (+1.2% and +1.2%, respectively) and the energy intensity of the economy decreased (−2.1%). When combined with changes in the fuel mix of the energy sector (−1.2%) and shifting consumption patterns (−0.2%), the net effect was a 2.1% decrease in emissions during 2011–2013 (Fig. 3).

Increases in the supply of natural gas affect two of the factors in our analysis: the fuel mix of the energy sector and, to a lesser extent, the energy intensity of the US economy. By decreasing gas prices, abundant gas encourages a shift in the fuel mix from more carbon-intensive coal to gas. In turn, a shift to gas may contribute to decreased energy intensity because gas-fired power plants are on average 20% more efficient at converting fuel energy to electricity than coal plants^12^.

The boom of natural gas from breakthroughs in hydraulic fracturing of shale deposits had only just begun to affect US gas supplies in 2009 (ref. 5). Thus, the decrease in emissions from changes in the fuel mix of the energy sector prior 2009 reflects an independent and longer-term trend of the declining use of coal in the US energy sector (see, for example, Fig. 2c). However, as seen in Fig. 3, changes in the US fuel mix from 2007 to 2009 alone would not have caused a decrease in US emissions.

Although the decreases in emissions since 2009 have been relatively small, the influence of shale gas is visible. For example, about half of the 2.1% decrease in emissions during 2011–2013 is related to changes in the fuel mix of the energy sector (−1.2%, orange bar in Fig. 3). Yet the decrease in the energy intensity of the US economy was nearly twice as strong an influence on emissions over the same period (purple bar in Fig. 3).

Although a drop in the energy intensity (exajoule per dollar output) of the energy sector in 2013 accounts for roughly a third of the observed decrease in US energy intensity in 2011–2013, the remaining two-thirds relate to changes in energy used by the transport and service sectors (Fig. 2a). Three unrelated trends underlie the decreasing energy intensity of these sectors. First, high gasoline prices during 2011–2013 (the average price of gasoline had remained above $3.40 per gallon during this period, in contrast to the average price of $2.50 per gallon in 2005) have contributed to both a reduction in per capita miles driven (Supplementary Fig. 1a) and an increase in average fuel efficiency of vehicles (Supplementary Fig. 1b), and thus a 33% decrease in US gasoline consumption during 2011–2013. Second, a mild winter in 2012 meant less energy was used for heating and thus reduced energy intensity of the service sector (households also used less energy for home heating, which accounts for part of the drop in consumption volume)^13^ (Supplementary Fig. 2). Last, there is evidence that manufacturing in the United States became more energy efficient: energy use by manufacturing was nearly constant 2011–2013 despite average annual growth in GDP of 2.3% per year over the same period.

Shifts in the production structure of the US economy between 2007 and 2013 have consistently exerted a downward influence on US emissions, as the volume and type of intermediate goods used by various industry sectors has evolved and become more efficient (blue bars in Fig. 3). Yet this structural shift also reflects the progressive offshoring of emissions-intensive industries to China and other developing countries over the analysed period^14^. For instance, between 2009 and 2011, when changes in domestic production structure exerted a downward influence on US CO_2_ emissions (−1%, blue bar in Fig. 3), we calculated that the net import of emissions embodied in US trade increased by 32% (Supplementary Fig. 3). Trade data for the 2011–2013 period is not yet available.

Between 2009 and 2013, the share of US consumption of manufactured goods increased relative to services (Fig. 2b), but the net effect of changes in consumption patterns was to decrease emissions (by 1.1% between 2009 and 2011 and by 0.2% between 2011 and 2013; green bars in Fig. 3). This result reveals that changes in the types of goods being consumed over time can have a significant impact on emissions^15^,^16^, and that it is not as simple as the balance of manufactured goods and services.

### Discussion

Between 1997 and 2007, US emissions grew steadily (0.7% per year) as increases related to population growth and consumption volume (per capita consumption) outpaced the downward influence of improving energy intensity, shifting consumption patterns and production structure and decarbonizing fuel mix.

The large decrease (9.9%) in US CO_2_ emissions between 2007 and 2009 was primarily the result of the economic recession, evidenced by large decreases in household consumption, energy-intensive capital expenditures and export (Figs 1, 3 and 4). The recessionary belt-tightening may also have contributed to the significant efficiency gains in production structure.

Since 2009, the slow recovery of the US economy has urged emissions backup, but has been closely balanced by decreases in energy intensity, especially in the transport, manufacturing and service sectors (Fig. 2a), as well as changes in the fuel mix of the energy sector. The net effect has been very little change in emissions; between 2009 and 2013; US emissions have decreased by an average of 0.2% per year. Contrary to conventional wisdom, our decomposition analysis shows that changes in the fuel mix of the energy sector (including those related to the shale gas boom) account for a relatively small portion of this decrease.

In addition to a more robust understanding of the factors influencing US emissions during 1997–2013, our analysis may be helpful in assessing the efficacy of different forces to reduce US emissions in the future. For example, the modest effect of changes in the fuel mix of the energy sector on emissions in recent years suggests that further increase in the use of natural gas may be of limited benefit in decreasing emissions. This is because barring technology-specific policies (for example, Renewable Portfolio Standards), recent studies have shown that gas does not substitute for coal only; growth of emission-free technologies such as solar, wind and nuclear energy is also limited while gas is cheap^17^,^18^. In these studies, future increases in natural gas use act to both reduce domestic coal use and slow the growth of renewable energy, resulting in little net change to cumulative CO_2_ emissions^17^,^19^,^20^,^21^. Moreover, CO_2_ emissions are not the only consideration; a growing number of studies also show that increased leakage of methane from new natural gas infrastructure can offset CO_2_ reductions relative to coal^22^,^23^. Third, decreases in residential gas prices (Supplementary Fig. 4) may lead to rebound effects if people spend some of the money they saved heating their home on carbon- and energy-intensive goods^24^. And finally, decreased domestic demand for coal has enabled an increase in US coal exports to eager and growing overseas markets. The US power sector consumed 170 million fewer metric tons of coal in 2013 than in 2007, during which period coal exports doubled even as coal prices rose (Supplementary Fig. 5). Although CO_2_ emissions from US coal burned elsewhere are generally attributed to the country where those emissions occur, the emissions nonetheless contribute to global climate change (and in fact less energy may be produced per unit of CO_2_ emissions when the coal is burned in countries with less-efficient power plants). For all these reasons, further increases in the use of natural gas in the United States may not have a large effect on global greenhouse gas emissions and warming.

Similarly, further emissions reductions due to decreases in energy intensity are not inevitable. As can be seen in Fig. 2a, the energy intensity of utilities increased between 2009 and 2013, perhaps because such utilities chose to pass the cost savings related to cheap gas along to their customers^25^. The energy intensity of other industry sectors also shows no long-term decreasing trend (Fig. 2a). In contrast, any gas-driven recovery of US manufacturing, such as in the production of vehicles and heavy machinery^26^, will tend to increase the average energy intensity of the US economy.

Sustaining economic growth while also drastically reducing emissions to the levels targeted by the Obama administration^27^ will depend upon large additional decreases in the energy intensity of the US economy as well as radical decarbonization of the energy sector (that is, very large changes in the fuel mix of the energy sector away from fossil fuels and toward renewables and/or nuclear energy). Although increased use of natural gas by the energy sector has helped to keep US CO_2_ emissions from rising during the economic recovery of 2009–2013, our decomposition analysis shows that decreases in the energy intensity of the manufacturing, transport and service sectors over the same period were even more important, and that the largest decrease in emissions was due to decreased consumption during the recession of 2007–2009. However, the recovering economy is now urging emissions backup, it is not clear whether decreases in energy intensity will continue, and the overall climate benefits of increased gas use are in question. Future reductions in US emissions will depend upon policies (for example, the Environmental Protection Agency Clean Power Plan) that can lock-in the recessionary emissions reductions and ensure continued decarbonization of the US energy system by deployment of more efficient and low-carbon energy technologies^28^.

## Methods

### Index decomposition versus structural decomposition

Index decomposition analysis (IDA) and SDA are two decomposition methods that have been frequently used to calculate the contribution of different factors to the overall change in carbon emissions and energy consumption. IDA is often used in studies that aim to understand the drivers of energy use and emissions in a specific economic sector, while SDA is used primarily by input–output practitioners whose research focus on the changes in energy consumption and emissions of a whole economy, for example, a country, a region, or the whole world^29^. Due to its simplicity, transparency and lower data requirements, the IDA approach based on index theory^30^,^31^,^32^,^33^ had been applied in numerous studies in the past^34^,^35^. However, these advantages of the IDA approach may mean limitations for more detailed in-depth analysis. For instance, lower data requirements also mean less detailed decomposition of economic production structure^34^ because the IDA approach cannot analyse the interdependency of different economic sectors^35^. Similarly, IDA does not distinguish intermediate and final consumption, and thus cannot capture indirect impacts of change in final consumption. In this study, we opt to use SDA based on input–output analysis^34^. The SDA overcomes many of the static features of input–output models, enabling the evaluation of changes over time in economic structure, final demand components and categories. The SDA is capable of distinguishing a range of production effects and final demand effects that the IDA approach lacks^35^, and allows assessment of both direct and indirect effects along the entire supply chain across upstream and downstream industries^36^. Although the high level of data requirement by the SDA approach has been a barrier in the past in light of the fact that many countries publish input–output tables only once every 5 or more years, the recent development of global time series input–output databases (for example, World Input–Output Database (WIOD)^37^ and The EOAR multi-region IO database^38^) and more regular publication of economic-structure data in countries like the United States now make time series SDA feasible. More detailed discussion on comparison of IDA and SDA approaches and their methodological developments can be found in Hoekstra and van den Bergh^34^ and Su and Ang^29^.

### Structural decomposition analysis

SDA is a quantitative methodology based on input–output modelling. SDA is a popular tool in assessing the contributions of different factors and industry sectors to changes in energy use and CO_2_ emissions over time. The method has been applied to many different countries such as Australia^39^, Denmark^40^,^41^, India^42^, Korea^43^, Netherlands^44^, the United States^45^ and China^15^,^35^,^46^,^47^.

Input–output analysis is an accounting procedure that relies on national or regional input–output tables. A country's input–output tables show the flows of goods and services and thus the interdependencies between suppliers and consumers along the production chain across upstream and downstream industries within an economy and between economies^48^. Environmental input–output analysis illustrates the economy-wide environmental repercussions (here we use CO_2_ emissions as environmental indicator) triggered by economic activity, and can be expressed mathematically as


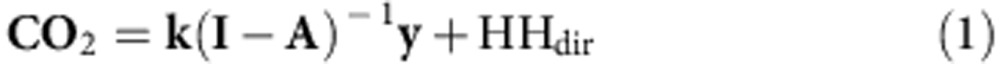


where CO_2_ is the total economy-wide CO_2_ emissions; **k** is a row vector of emission coefficients (emissions per unit of economic output) in each economic sector; **I** is the identity matrix; **A** is a matrix, and each column of **A** shows input requirement from each sector to produce one unit output of this column sector; **y** is a column vector of final consumption; HH_dir_ is a scalar of household direct emissions, for example, heating and driving. We consider the production structure through **L=(I–A)**^**−1**^, which is the renowned Leontief inversion matrix. Changes in the production structure thus refer to changing input requirements of each sector or, in other words, industries using more or less intermediate inputs from each other. It has been widely discussed that both emissions per unit of energy consumption (fuel mix) and energy efficiency (energy consumption per unit of economic output) are vital to the emission intensity of an economy^49^,^50^. Hence, we further decompose the emission coefficients, **k** into emission intensity (emissions per unit of energy consumption) and energy intensity (energy consumption per unit of output) **k**=**fÊ**, where **f** is a row vector of emissions per unit of energy use (fuel mix) and **Ê** is the diagonalized matrix of energy use per unit of economic output. To distinguish the contributions of different final demand components, we further decompose **y** into three components—average consumption structure, per capita consumption volume and population: **y=y**_**s**_*y*_*v*_ *p*, where **y**_**s**_ is a vector of per capita consumption patterns; *y*_*v*_ is a scalar of per capita consumption volume; *p* is a scalar of population which could appear at the front or the back of the input–output equation. Therefore, equation (1) can be transformed to:


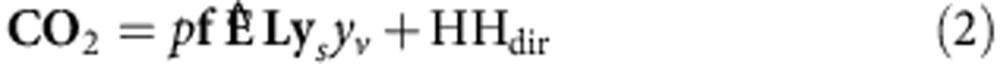


Over a given period of time, any changes in CO_2_ emissions in a country can be represented by equation (2), in which the seven factors of population, fuel mix, energy intensity, production structure, consumption patterns and consumption volume, plus household direct emissions, fully account for the changes in CO_2_ emissions. A total difference of equation (2) generates equation (3)


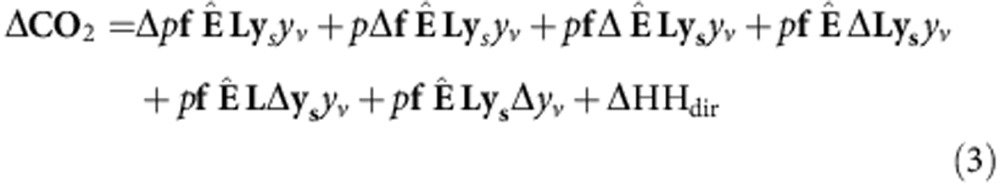


where, Δ is the difference operator. Equation (3) converts six multiplicative terms in the first term of equation (2) into six additive terms. Each additive term in equation (3) represents the contribution to a change in CO_2_ emissions triggered by a factor assuming all other factors are constant. For example, in the sixth term, Δ**y**_**v**_ is change in per capita consumption volume, and the term represents the change of total CO_2_ emission caused by a change in per capita consumption volume, with population size, fuel mix, energy intensity, production structure and consumption patterns staying constant.

In the SDA, it is possible to compare different terms relative to any time point within a study period. However, there is no unique solution for the decomposition. In this study, we use the average of all possible first-order decompositions suggested by Dietzenbacher and Los^51^ and Seibel^52^ (see Supplementary Methods and Supplementary Table 1 for a detailed discussion). We also simplify the presented results by combining direct CO_2_ emissions from households (for example, natural gas heating in homes) with the emissions embodied in consumed goods (that is, ‘consumption volume').

The US input–output tables from 1997 to 2013 were collected from the Bureau of Economic Analysis which is in make-use format^11^. We convert the make-use table to symmetric input–output table following the method by Miller and Blair^48^ and then aggregated them into 35 economic sectors to match the energy and emission data from the WIOD^37^.

The CO_2_ emissions and energy data from 2010 to 2013 were collected from the US Energy Information Administration (EIA)^2^. EIA only publishes energy and emission data at aggregate sectoral level including manufacturing, electric power, commercial and residential sectors. We disaggregated energy use of these four sectors into 35 economic sectors according to the sectoral energy purchase collected from Bureau of Economic Analysis^11^. Also, for data consistency, we scale the energy and CO_2_ emission data from WIOD to match the EIA data.

Our analysis focuses on US fossil fuel CO_2_ emissions and does not include emissions of non-CO_2_ greenhouse gases such as methane. Incorporating methane in the analysis would tend to reduce the climate benefit of gas via the fuel mix of the power sector because of fugitive methane emissions, which may be substantial^23^,^53^. Our analysis also focuses on CO_2_ emissions produced in the United States; emissions embodied in imports from other countries are not included. This territorial perspective is consistent with the focus of prospective policies, although some analysts have argued for consumption-based accounting as a basis for climate policy^54^,^55^,^56^. For this reason, we also pay attention to how changes in trade may have affected the factors of US production structure and energy intensity by offshoring of energy-intensive manufacturing^57^.

### Decomposing final demand

Because changes in the volume of goods and services consumed were the single most important influence on US emissions between 1997 and 2013, we also analysed four separate components of final demand to assess the trends in emissions related to each category as well as the important influences on emissions in each case. Figure 4 shows the emissions associated with different final demand (consumption) components: household consumption (Fig. 4a), governmental expenditure (Fig. 4b), capital formation (Fig. 4c) and exports (Fig. 4d).

Between 2007 and 2013, emissions associated with household consumption decreased by 11.0%, which was almost entirely driven by changes in fuel mix and production structure, especially between 2009 and 2013, since consumption volume was constant (Fig. 4a). Emissions associated with government expenditures in the same time period decreased by 4.8%, and it was largely driven by changes in energy intensity and production structure (Fig. 4b). In contrast, emissions related to capital formation decreased by 24.4% between 2007 and 2013, primarily due to a huge decline in the volume of capital investment (Fig. 4c, red curve). However, changes in emissions related to exports between 2007 and 2013 were almost entirely the result of changes in the volume of exports, with the other factors cancelling each other out (Fig. 4d).

## Additional information

**How to cite this article:** Feng, K. *et al.* Drivers of the US CO_2_ emissions 1997–2013. *Nat. Commun.* 6:7714 doi: 10.1038/ncomms8714 (2015).

## Supplementary Material

Supplementary Information Supplementary Figures 1-5, Supplementary Table 1, Supplementary Methods and Supplementary References
