# Supplementary material for: Drivers of the US CO2 emissions 1997–2013
Source: Nat Commun. 2015 Jul 21;6:7714. doi: 10.1038/ncomms8714 (PMC4518269; doi:10.1038/ncomms8714)
Supplement: Supplementary Information — Supplementary Figures 1-5, Supplementary Table 1, Supplementary Methods and Supplementary References [file ncomms8714-s1.pdf]

Supplementary Information

Supplementary Figure 1

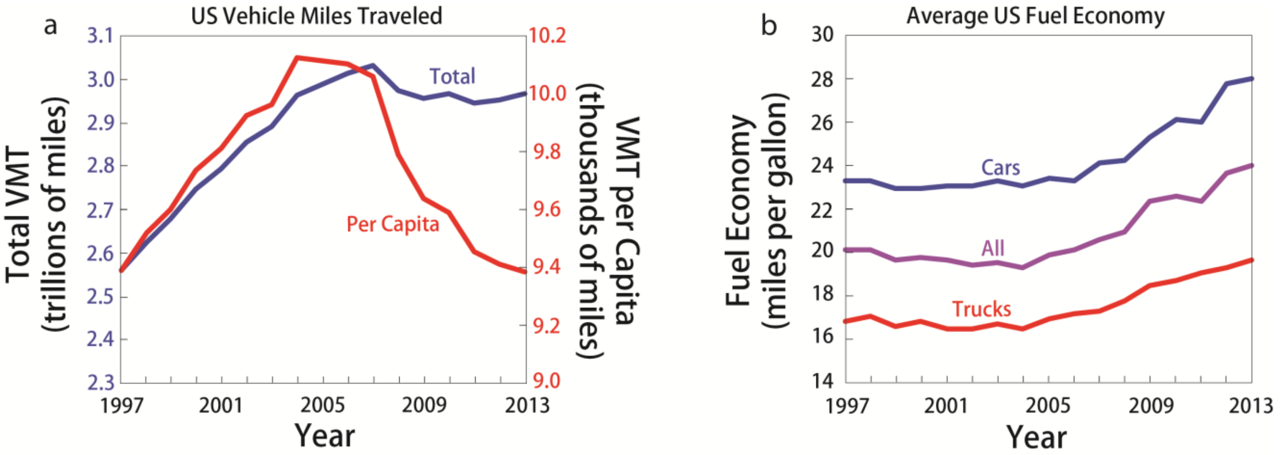

Supplementary Figure 1. Trends affecting the energy intensity of road transport in the US 1997-2013. Per capita vehicle miles traveled decreased sharply between 2007 and 2013 (**A**, red curve) [1] and the average fuel economy of vehicles increased substantially over the same period (**B**) [2].

Supplementary Figure 2

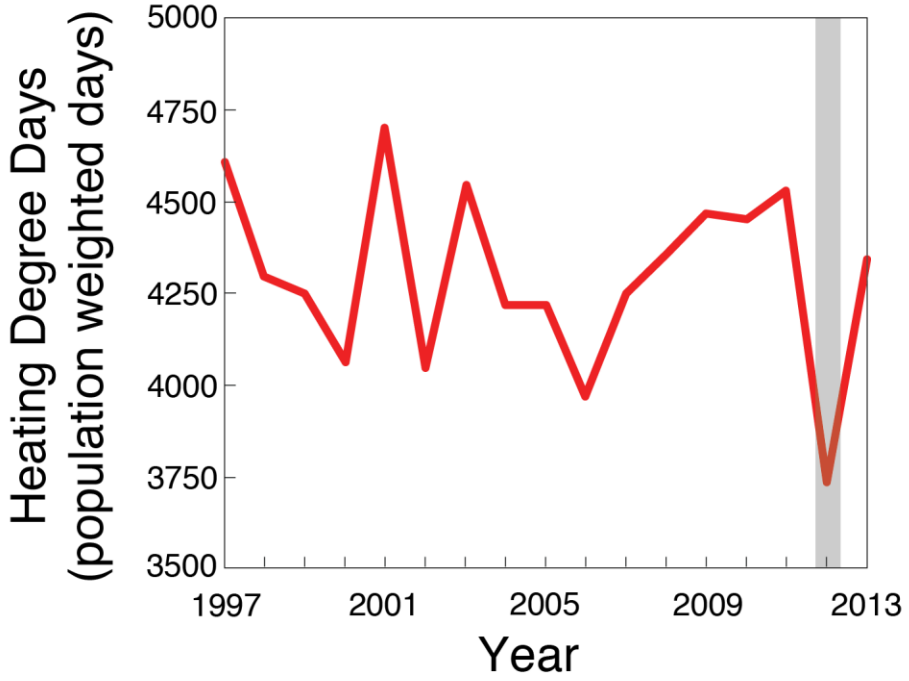

Supplementary Figure 2. Trend in population-weighted heating degree days 1997-2013 [3]. The mild winter of 2011-2012 resulted in the lowest population-weighted heating degree days over the entire period.

Supplementary Figure 3

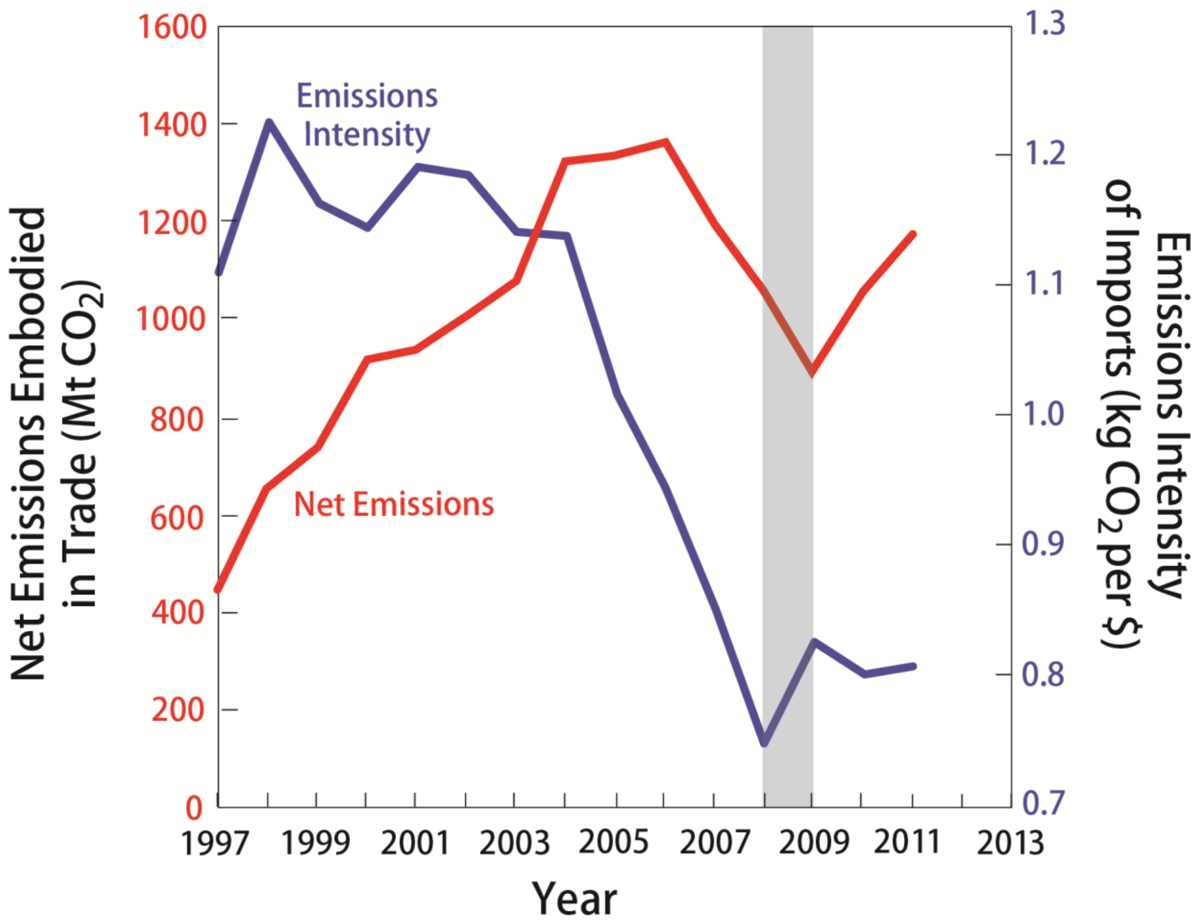

Supplementary Figure 3. Trends in traded emissions. The red curve shows net emissions embodied in trade 1997-2011 (emissions embodied in imports less emissions embodied in exports) [4]. The blue curve shows the emissions intensity of imports over the same period. Data for the years 2012-2013 are not yet available. Net import of emissions fell during the global economic recession (gray shading), but the emissions intensity of the goods that were being imported jumped up in response to changes in the production structure of US industry.

Supplementary Figure 4

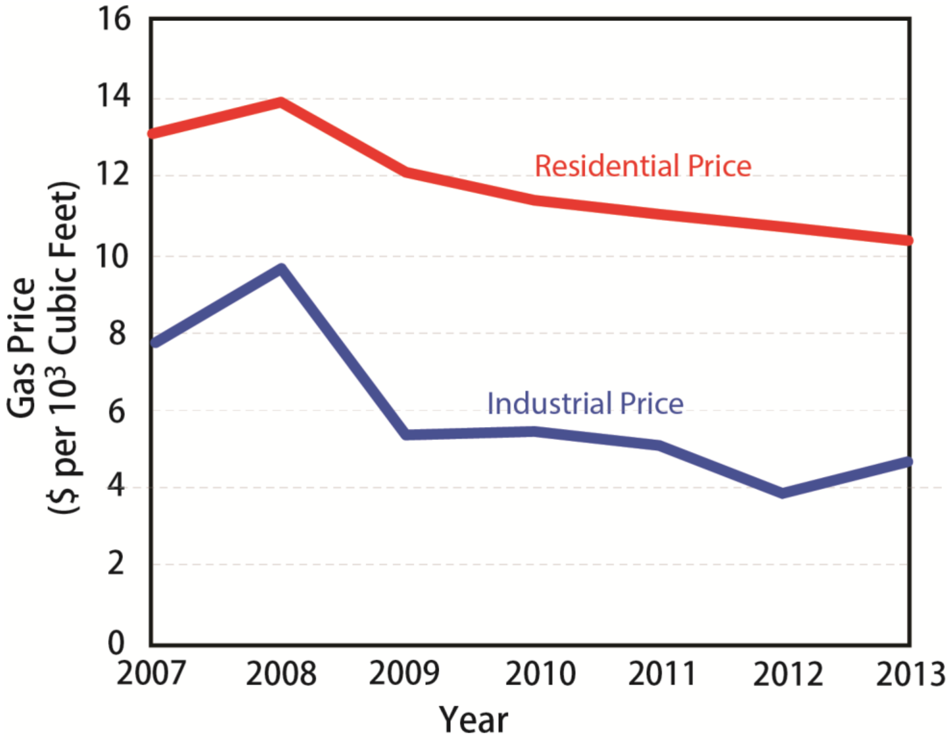

Supplementary Figure 4. Prices of natural gas 2007-2013. –Prices in US dollars per 1000 Cubic Feet [5].

Supplementary Figure 5

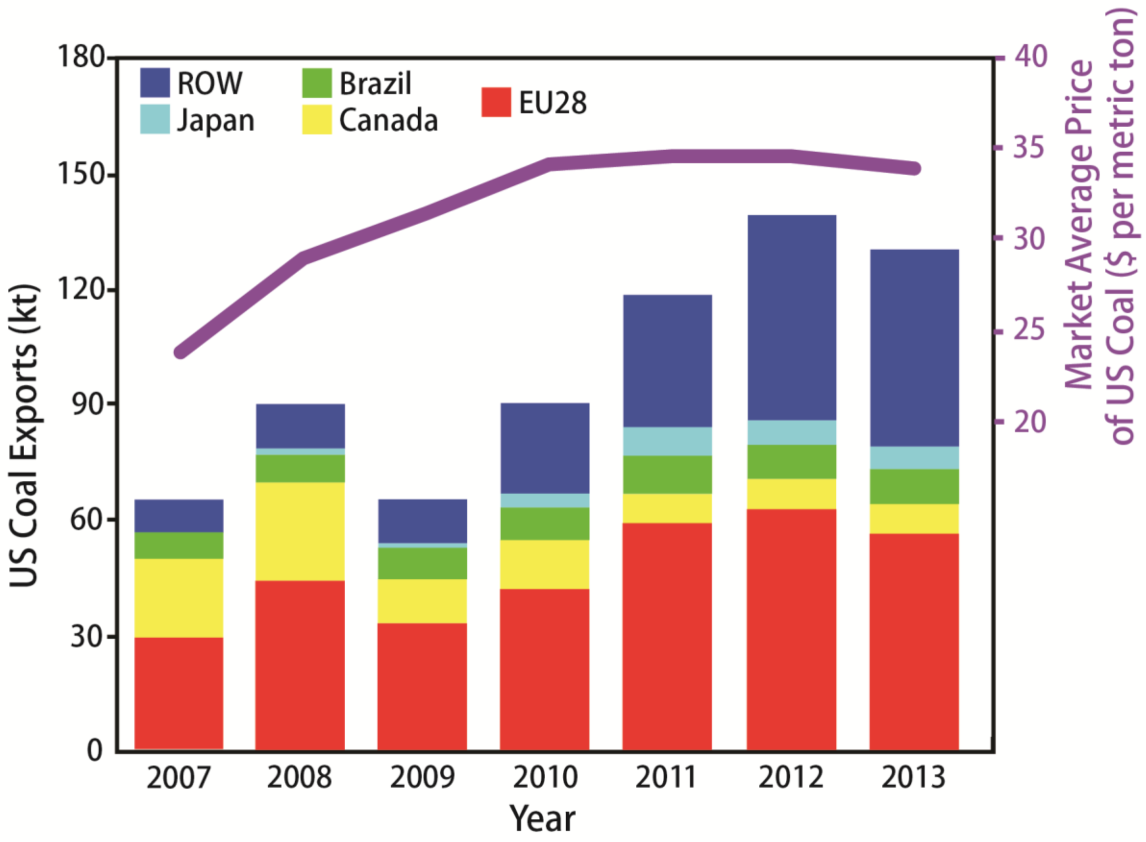

Supplementary Figure 5. US coal export from 1995 to 2013 by destination region. Exports shown in metric tons [5].

Supplementary Table 1

K	Subscript for the components in the coefficients					Weight
	first	second	third	fourth	fifth	
**0**	*t* - 1	*t* - 1	*t* - 1	*t* - 1	*t* - 1	**120**
**1**	*t*	*t* - 1	*t* - 1	*t* - 1	*t* - 1	**24**
	t - 1	*t*	*t* - 1	*t* - 1	*t* - 1	
	t - 1	*t* - 1	*t*	*t* - 1	*t* - 1	
	t - 1	*t* - 1	*t* - 1	*t*	*t* - 1	
	t - 1	*t* - 1	*t* - 1	*t* - 1	*t*	
**2**	*t*	*t*	*t* - 1	*t* - 1	*t* - 1	**12**
	t	*t* - 1	*t*	*t* - 1	*t* - 1	
	t	*t* - 1	*t* - 1	*t*	*t* - 1	
	t	*t* - 1	*t* - 1	*t* - 1	*t*	
	t - 1	*t*	*t*	*t* - 1	*t* - 1	
	t - 1	*t*	*t* - 1	*t*	*t* - 1	
	t - 1	*t*	*t* - 1	*t* - 1	*t*	
	t - 1	*t* - 1	*t*	*t*	*t* - 1	
	t - 1	*t* - 1	*t*	*t* - 1	*t*	
	t - 1	*t* - 1	*t* - 1	*t*	*t*	
**3**	*t*	*t*	*t*	*t* - 1	*t* - 1	**12**
	t	*t*	*t* - 1	*t*	*t* - 1	
	t	*t*	*t* - 1	*t* - 1	*t*	
	t	*t* - 1	*t*	*t*	*t* - 1	
	t	*t* - 1	*t*	*t* - 1	*t*	
	t	*t* - 1	*t* - 1	*t*	*t*	
	t - 1	*t*	*t*	*t*	*t* - 1	
	t - 1	*t*	*t*	*t* - 1	*t*	
	t - 1	*t*	*t* - 1	*t*	*t*	
	t - 1	*t* - 1	*t*	*t*	*t*	
**4**	*t*	*t*	*t*	*t*	*t* - 1	**24**
	t	*t*	*t*	*t* - 1	*t*	
	t	*t*	*t* - 1	*t*	*t*	
	t	*t* - 1	*t*	*t*	*t*	
	t - 1	*t*	*t*	*t*	*t*	
5	*t*	*t*	*t*	*t*	*t*	**120**

Supplementary Table 1. Subscripts for the components of "Δfactor's" coefficients and their weights

Source: Modified from Rørmose and Olsen (2005) [6]

Supplementary Methods

As presented in Methods section in the main text, in this study the change of CO_2 emission is decomposed into six additive terms, and each term represents the contribution of the changing factor to the total change of CO_2 emission in the US. One can perceive a logical pattern that the changing factors is placed, at each term, in turn from left to right in the product with all other factors; and the other unchanged factors on the left hand side of the changing factors are in base year value (year "$t - 1$"); and the ones on the right hand side of the changing factors are in target year value (year "t"). Therefore, by extracting the unchanged values in each term the equation can be merged as:

$$\Delta \mathbf{CO_2} = \mathbf{w}^{p}\boldsymbol{\Delta}p + \mathbf{w}^{f}\Delta\mathbf{f} + \mathbf{w}^{E}\Delta\mathbf{E} + \mathbf{w}^{L}\Delta\mathbf{L} + \mathbf{w}^{y_s}\Delta\mathbf{y}_s + \mathbf{w}^{y_v}\Delta y_v \qquad \textbf{(1)}$$

where the w^{p}, w^{f}, w^{E}, w^{L}, w^{y_s}, and w^{y_v} are the so-called "weight" or "coefficient" for each "Δfactor" respectively. The calculation of these "weights" or "coefficients" are usually done via econometric methods; alternatively, they can be generated via a more straight forward way by deriving them with the structural decomposition method [7, 8].

However, supplementary equation (1) is not a unique decomposition equation, which is only one of the 720 decomposition equations by assuming the order of the driving forces of "$\mathbf{p}\cdot\mathbf{f}\cdot\mathbf{E}\cdot\mathbf{L}\cdot y_s\cdot y_v$". However, the order can also be "$\mathbf{f}\cdot\mathbf{p}\cdot\mathbf{E}\cdot\mathbf{L}\cdot\mathbf{y}_s\cdot y_v$" or "$\mathbf{f}\cdot\mathbf{E}\cdot\mathbf{p}\cdot\mathbf{L}\cdot\mathbf{y}_s\cdot y_v$" and so on. Although each decomposition equation would produce exactly the same result for ΔCO_2, de Haan [9] found that the size of the contribution of each "Δfactor" significantly differs across the equations. In other words, the "coefficient" (w) of each "Δfactor" is varied in different equations.

Due to the non-uniqueness issue, Dietzenbacher and Los [10] suggested to take the average of all the $n!$ ($6!$ in this case) decomposition equations (Supplementary Table 1). In order to do so, all the 720 equations need to be sorted into a standard order, for example, every term in the equation needs to be re-arranged to the order of "$\mathbf{p}\cdot\mathbf{f}\cdot\mathbf{E}\cdot\mathbf{L}\cdot\mathbf{y}_s\cdot y_v$", and the "Δfactor" is in turn placed from the first factor of "p" in the first term of the equation to the last factor of y_s in the last (seventh) term. Then, all the equations have been re-arranged in the same pattern. For example, the first term of every equation contains the information of the contribution of population growth (Δp) to the change of CO_2 (ΔCO_2) with other factors kept unchanged. The product of the unchanged values of other factors is the "coefficient" for Δp. The "coefficient" $\mathbf{f}_{(t-1)} \cdot \mathbf{E}_{(t-1)} \cdot \mathbf{L}_{(t-1)} \cdot \mathbf{y}_{s(t-1)} \cdot y_{v(t-1)}$ appears 120 times, and same as the "coefficient" $\mathbf{f}_t \cdot \mathbf{E}_t \cdot \mathbf{L}_t \cdot \mathbf{y}_{st} \cdot y_{vt}$ does. de Haan [9] and Seibel [11]

found that each term in the equation always has $2^{(n-1)}$ different "coefficients" attached to the "Δfactor", $2^{(6-1)} = 32$ different "coefficients" to every "Δfactor" in this case.

Next one can calculate the "weights" of the "coefficients" which is attached to the "Δfactor". The easiest way is via observations, to count how many cases of "Δfactor" are attached to the same "coefficient". For example as mentioned previously, the "coefficient" $\mathbf{f}_{(t-1)} \cdot \mathbf{E}_{(t-1)} \cdot \mathbf{L}_{(t-1)} \cdot \mathbf{y}_{s(t-1)} \cdot \mathbf{y}_{v(t-1)}$ appears 120 times in the 720 equations, and therefore its weight is 120. However, the observation method could be difficult in large number of decomposition equations with more than 5 factors.

Seibel [11] proposed a mathematic method to deal with this. Firstly, let k represent the number of subscript "$t - 1$" (base year) in a coefficient; k runs from "0" to "$n - 1$"; therefore, the number of subscript "t" (target year) would be "$n - 1 - k$". Secondly, for each k, the number of different coefficients attached to the "Δfactor" can be calculated by:

$$\frac{(n-1)!}{(n-1-k)! \cdot k!} \qquad \textbf{(2)}$$

In this study, n is set to 6 (six factors). So when k = 0 or 5, there is only one coefficient for each case; when k = 1 or 4, the number of different coefficients are 5 respectively; when k = 2 or 3, there would be 10 different coefficients. Thirdly, supplementary equation (3) calculates how many times each of these coefficients is repeated as "weights" for each "Δfactor" term in every equation of $n!$. The results for supplementary equations (2) and (3) are shown in Supplementary Table 1 for the case of n = 6

$$(n-1-k)! \cdot k! \qquad \textbf{(3)}$$

Therefore, each "w" attached to the "Δfactor" in supplementary equation 1 can be present, for example,

$$\begin{aligned} \mathrm{w}^{\mathrm{p}}\Delta \mathrm{p} = \tfrac{1}{720}[&(120 \cdot \Delta p \cdot \mathbf{f}_{(t-1)} \cdot \mathbf{E}_{(t-1)} \cdot \mathbf{L}_{(t-1)} \cdot \mathbf{y}_{\mathbf{s}(t-1)} \cdot y_{v(t-1)}) + \\ &(24 \cdot \Delta p \cdot \mathbf{f}_{t} \cdot \mathbf{E}_{(t-1)} \cdot \mathbf{L}_{(t-1)} \cdot \mathbf{y}_{s(t-1)} \cdot y_{v(t-1)}) + \\ &(24 \cdot \Delta p \cdot \mathbf{f}_{(t-1)} \cdot \mathbf{E}_{t} \cdot \mathbf{L}_{(t-1)} \cdot \mathbf{y}_{s(t-1)} \cdot y_{v(t-1)}) + \\ &(24 \cdot \Delta p \cdot \mathbf{f}_{(t-1)} \cdot \mathbf{E}_{(t-1)} \cdot \mathbf{L}_{t} \cdot \mathbf{y}_{s(t-1)} \cdot y_{v(t-1)}) + \\ &(24 \cdot \Delta p \cdot \mathbf{f}_{(t-1)} \cdot \mathbf{E}_{(t-1)} \cdot \mathbf{L}_{(t-1)} \cdot \mathbf{y}_{st} \cdot y_{v(t-1)}) + \\ &(24 \cdot \Delta p \cdot \mathbf{f}_{(t-1)} \cdot \mathbf{E}_{(t-1)} \cdot \mathbf{L}_{(t-1)} \cdot \mathbf{y}_{s(t-1)} \cdot y_{vt}) + \end{aligned}$$

$$\left(12 \cdot \Delta p \cdot \mathbf{f}_t \cdot \mathbf{E}_t \cdot \mathbf{L}_{(t-1)} \cdot \mathbf{y}_{s(t-1)} \cdot y_{v(t-1)}\right) +$$
$$\left(12 \cdot \Delta p \cdot \mathbf{f}_t \cdot \mathbf{E}_{(t-1)} \cdot \mathbf{L}_t \cdot \mathbf{y}_{s(t-1)} \cdot y_{v(t-1)}\right) +$$
$$\cdots$$
$$\left(12 \cdot \Delta p \cdot \mathbf{f}_{(t-1)} \cdot \mathbf{E}_t \cdot \mathbf{L}_{(t-1)} \cdot \mathbf{y}_{st} \cdot y_{vt}\right) +$$
$$\left(12 \cdot \Delta p \cdot \mathbf{f}_{(t-1)} \cdot \mathbf{E}_{(t-1)} \cdot \mathbf{L}_t \cdot \mathbf{y}_{st} \cdot y_{vt}\right) +$$
$$\left(24 \cdot \Delta p \cdot \mathbf{f}_t \cdot \mathbf{E}_t \cdot \mathbf{L}_t \cdot \mathbf{y}_{st} \cdot y_{v(t-1)}\right) +$$
$$\left(24 \cdot \Delta p \cdot \mathbf{f}_t \cdot \mathbf{E}_t \cdot \mathbf{L}_t \cdot \mathbf{y}_{s(t-1)} \cdot y_{vt}\right) +$$
$$\left(24 \cdot \Delta p \cdot \mathbf{f}_t \cdot \mathbf{E}_t \cdot \mathbf{L}_{(t-1)} \cdot \mathbf{y}_{st} \cdot y_{vt}\right) +$$
$$\left(24 \cdot \Delta p \cdot \mathbf{f}_t \cdot \mathbf{E}_{(t-1)} \cdot \mathbf{L}_t \cdot \mathbf{y}_{st} \cdot y_{vt}\right) +$$
$$\left(24 \cdot \Delta p \cdot \mathbf{f}_{(t-1)} \cdot \mathbf{E}_t \cdot \mathbf{L}_t \cdot \mathbf{y}_{st} \cdot \mathbf{y}_{vt}\right) +$$
$$(120 \cdot \Delta p \cdot \mathbf{f}_t \cdot \mathbf{E}_t \cdot \mathbf{L}_t \cdot \mathbf{y}_{st} \cdot y_{vt})$$

and it is similar to obtain other "*w*"s in supplementary equation (1).

Supplementary References

1. Historical Monthly VMT Report. (U.S. Department of Transportation FHA) (2013).

2. EPA. Light-Duty Automotive Technology, Carbon Dioxide Emissions, and Fuel Economy Trends: 1975 - 2013. United States Environmental Protection Agency (2013).

3. NOAA. Degree Days Statistics. (Center NWSCP) (2014).

4. WIOD. World Input-Output Database. the 7th Framework Programme, the European Commission (2012).

5. EIA. *International Energy Statistics*. U.S. Energy Information Administration (2014).

6. Rørmose P, Olsen T. Structural Decomposition Analysis of Air Emissions in Denmark 1980-2002. In: *15th International Input-Output Conference* (2005).

7. Hoekstra R, van der Bergh JCJM. Structural decomposition analysis of physical flows in the economy. *Environmental and Resource Economics* **23**, 357--378 (2002).

8. Guan D, Peters GP, Weber CL, Hubacek K. Journey to world top emitter: An analysis of the driving forces of China's recent CO_2 emissions surge. *Geophysical Research Letters* **36**, 1-5 (2009).

9. de Haan M. A Structural Decomposition Analysis of Pollution in the Netherlands. *Economic Systems Research* **13**, 181-196 (2001).

10. Dietzenbacher E, Los B. Structural decomposition techniques: Sense and sensitivity. *Economic Systems Research* **10**, 307--323 (1998).

11. Seibel S. Decomposition Analysis of Carbon Dioxide Emission Changes in Germany - Conceptual Framework and Empirical Results. European Commission. In: *Working Paper* (2003).
